# Supplementary material for: Effect of Neutron Radiation on 10BPA-Loaded Melanoma Spheroids and Melanocytes
Source: Cells. 2025 Feb 6;14(3):232. doi: 10.3390/cells14030232 (PMC11816858; doi:10.3390/cells14030232)
Supplement: Supplementary file 1 [file cells-14-00232-s001.zip › cells-3343222-supplementary.pdf]

cells

MDPI

Supplementary Material S1. Viability of Melanocytes and Melanoma Cells After Incubation with BPA

The 2D cell cultures, both melanoma cell lines, FM55p and WM266-4, and melanocytes (HEMa-LP) were incubated with 50 µg B/mL for 2 h, 4 h, 6 h, and 12 h. The control group consisted of cells incubated with standard growth medium. After the incubation, cells were washed three times with PBS w/o Ca^{2+}, Mg^{2+} and harvested by trypsinization. Trypsin was inactivated by a medium with serum (Trypsin Neutralizer solution was used for HEMa-LP) after cell detaching. Melanoma cells were centrifuged at 260 g for 10 min and melanocytes were centrifuged at 180 g for 7 min. Pellets of cells were resuspended in fresh medium and counted with trypan blue by automatic cell counter LUNA II. Results are presented in Table S1.

Table S1. Results of viability test for melanoma cells (FM55p and WM266-4) and melanocytes (HEMa-LP) after 2h, 4h, 6h, and 12h incubation with BPA, and for control samples for each cell line (not incubated with BPA). Viability was calculated as the ratio of the number of alive cells to the total number of cells (alive and dead) and presented in percentage. The presented data are the averages of results obtained with three repetitions and are given with the standard deviation (SD).

Cell Line	HEMa-LP	FM55p	WM266-4
Time of Incubation with BPA [h]	Viability [%]	Viability [%]	Viability [%]
2	96.6 ± 0.3	97.6 ± 0.4	97.9 ± 0.3
4	95.7 ± 1.5	97.7 ± 0.2	97.6 ± 0.6
6	96.6 ± 0.4	97.3 ± 0.5	98.3 ± 0.4
12	97.1 ± 0.1	98.5 ± 0.1	98.6 ± 0.2
Control	98.2 ± 0.9	97.8 ± 0.7	98.8 ± 0.1

Spheroids formed from both cell lines, FM55p and WM266-4, were cultured in low-adhesive plates using 2000 cells per well. On the 7th day, spheroids were incubated for an appropriate time with BPA 50 µg B/mL (2 h, 4 h, 6 h, and 12 h). After BPA incubation, spheroids were collected into 50 mL tubes and centrifuged. Next, they were washed three times with PBS w/o Ca^{2+}, Mg^{2+} and dissociated with trypsin-EDTA incubation for 15 min. in incubator (37 °C) and washed with a medium with serum and centrifuged to gain pellets of cells. In the next step, cells were resuspended in 200 µL of fresh medium and counted after staining with trypan blue by the automatic cell counter LUNA II. Results are presented in Table S2.

Table S2. Viability of spheroids formed from the FM55p and WM266-4 cell lines after 2 h, 4 h, 6 h, and 12 h incubation with BPA. Viability was calculated as the registered fraction of alive cells in the spheroid and presented in percentage. The shown data represent average values calculated with the results of three repetitions and are given with standard deviation.

Cell Line	FM55p	WM266-4
Time of Incubation with BPA [h]	Viability [%]	Viability [%]
2	97.8 ± 1.2	99.8 ± 0.2
4	97.8 ± 2.2	99.8 ± 0.2
6	98.9 ± 0.2	99.8 ± 0.2
12	99.3 ± 0.7	98.8 ± 0.4
Control	99.7 ± 0.3	99.5 ± 0.1

Supplementary Material S2. Results of Student's *t*-Test (*p*-Value) Comparing the Outcomes of the Comet Assay

(a) 2D cell cultures

HEMa-LP Cell Line	Irradiated with Neutrons After BPA Incubation (IR_B)	Irradiated with Neutrons (IR)
**Time After Irradiation**	**Control vs. 2Gy**	**Control vs. 2 Gy**
1 h	0.0002	0.00697
24 h	ns *	ns *
1 h: IR_B vs. IR	<0.0001	
24 h: IR_B vs. IR	ns *	

* not significant.

FM55p Cell Line	Irradiated with Neutrons After BPA Incubation (IR_B)	Irradiated With Neutrons (IR)
**Time after radiation**	**Control vs. 2 Gy**	**Control vs. 2 Gy**
1 h	<0.0001	ns *
24 h	0.0013	ns *
1h: IR_B vs. IR	0.0001	
24h: IR_B vs. IR	0.0073	

* not significant.

WM266-4 Cell Line	*p*-Value IR_B	*p*-Value IR
**Time After Radiation**	**Control vs. 2 Gy**	**Control vs. 2 Gy**
1 h	ns *	ns *
24 h	ns *	ns *
1 h: IR_B vs. IR	ns *	
24 h: IR_B vs. IR	ns *	

* not significant.

(b) 3D cell cultures

FM55p Cell Line	*p*-Value IR_B			*p*-Value IR			*p*-Value	
**Time After Irradiation**	**Control vs. 2 Gy**	**Control vs. 6 Gy**	**2 Gy Vs. 6 Gy**	**Control vs. 2 Gy**	**Control vs. 6 Gy**	**2 Gy Vs. 6 Gy**	**IR_B vs. IR 2 Gy**	**IR_B vs. IR 6 Gy**
1 h	0.011	0.001	0.001	0.001	0.006	0.010	0.001	<0.0001
24 h	ns *	0.002	0.038	0.008	-	-	0.028	-
48 h	ns *	ns *	ns *	ns *	-	-	0.002	-

* not significant.

WM266-4 Cell Line	*p*-Value IR_B			*p*-Value IR			*p*-Value	
**Time After Irradiation**	**Control vs. 2 Gy**	**Control vs. 6 Gy**	**2 Gy vs. 6 Gy**	**Control vs. 2 Gy**	**Control vs. 6 Gy**	**2 Gy vs. 6 Gy**	**IR_B vs. IR 2 Gy**	**IR_B vs. IR 6 Gy**
1 h	0.003	<0.0001	0.006	ns *	ns *	ns *	0.002	<0.0001
24 h	ns *	0.001	0.006	ns *	ns *	0.012	ns *	ns *
48 h	0.032	0.002	<0.0001	0.004	ns *	0.001	0.003	0.001

* not significant.

Supplementary Material S3. Results of Student's *t*-Test (*p*-Value) Comparing the Outcomes of the Proliferation Assay—Ki67 Protein Level

(a) FM55p cell line spheroids

	p-Value IR_B			*p*-Value IR			*p*-Value	
**Time After Irradiation**	**Control vs. 2 Gy**	**Control vs. 6 Gy**	**2 Gy vs. 6 Gy**	**Control vs. 2 Gy**	**Control vs. 6 Gy**	**2 Gy Vs. 6 Gy**	**IR_B vs. IR 2 Gy**	**IR_B vs. IR 6 Gy**
1 h	0.03	ns *	ns *	ns *	0.04	ns *	ns *	ns *
24 h	ns *	ns *	ns *	ns *	-	-	ns *	-
48 h	ns *	0.04	ns *	ns *	-	-	ns *	-

* not significant.

(b) WM266-4 cell line spheroids

	p-Value IR_B			*p*-Value IR			*p*-Value	
**Time After Irradiation**	**Control vs. 2 Gy**	**Control vs. 6 Gy**	**2 Gy vs. 6 Gy**	**Control vs. 2 Gy**	**Control vs. 6 Gy**	**2 Gy Vs. 6 Gy**	**IR_B vs. IR 2 Gy**	**IR_B vs. IR 6 Gy**
1 h	ns *	0.009	ns *	ns *	ns *	ns *	ns *	ns *
24 h	0.005	<0.0001	0.030	ns *	0.001	ns *	ns *	<0.0001
48 h	0.010	0.001	0.008	0.001	ns *	0.020	ns *	0.001

* not significant.
